# Nutritional and antioxidative properties of black goat meat cuts

**DOI:** 10.5713/ajas.18.0951

**Published:** 2019-03-07

**Authors:** Hye-Jin Kim, Hee-Jin Kim, Aera Jang

**Affiliations:** 1 Department of Animal Life Science, College of Animal Life Science, Kangwon National University, Chuncheon 24341, Korea

**Keywords:** Black Goat Meat, Bioactive Compound, Antioxidant Activity, Nutritional Value, Mineral

## Abstract

**Objective:**

In this study, we evaluated the nutritional value and antioxidant activity of black goat loin (BGL) and black goat rump (BGR) meat.

**Methods:**

We evaluated the proximate compositions, collagen and mineral contents, and fatty acid compositions of BGL and BGR with respect to their nutritional value. The levels of bioactive compounds such as L-carnitine, creatine, creatinine, carnosine, and anserine were also measured. The ferric reducing antioxidant power (FRAP), 2,2-azinobis (3-ethyl-benzothiazoline-6-sulfonic acid) (ABTS) radical scavenging, and oxygen radical absorption capacity (ORAC) were assessed to evaluate the antioxidant activity of BGL and BGR.

**Results:**

BGR showed higher collagen, Fe, Ca, P, and Na contents than did BGL (p<0.05). Notably, the Ca/P ratio was high in both BGR and BGL (1.82 and 1.54, respectively), thus satisfying the recommendation that the Ca/P ratio is between 1 and 2. BGL showed a significantly higher content of desirable fatty acids (stearic acid and total unsaturated fatty acids) than did BGR. In addition, the levels of creatine, carnosine, and anserine in BGL were higher than those in BGR (p<0.05). There was no significant difference in the antioxidant activity between BGL and BGR, as assessed by FRAP (both 15.92 μmol Trolox equivalent [TE]/g of dry matter [DM]), ABTS (12.51 and 12.90 μmol TE/g DM, respectively), and ORAC (101.25 and 99.06 μmol TE/g DM, respectively) assays.

**Conclusion:**

This was a primary study conducted to evaluate the differences in nutritional value and antioxidant activity between loin and rump cuts of black goat meat. Our results provide fundamental knowledge that can help understand the properties of black goat meat.

## INTRODUCTION

Goats have been together with humans for the consumption since the beginning of human civilization [1]. The Korean native goat (*Capra hircus coreanae*) is a breed of goats indigenous to Korea; approximately 80% of the population is predominantly black. Archaeological evidence on the origin of the Korean black goat is not available. However, historical literature indicates that the history of goat farming on the Korean Peninsula may date back over 2,000 years [2]. Korean black goats have been raised in the mountains on agricultural byproducts and native grass. With the recent increase in consumption of black goat meat, full-time farming of black goats has also increased in Korea [3].

Goat meat tends to be dark red in color and possesses a rough texture, with a flavor and aroma perceptibly different from those of lamb and mutton [4]. Goat meat is widely consumed in Asia and developing countries. Black goats have a low carcass weight, and the meat has a strong wild flavor. Most Korean people consume black goat meat in the form of meat and bone extracts rather than the meat itself. The extract of black goat meat has been considered a folk medicine and is used to improve the health of young children, pregnant women, and the elder population. The health benefits of these extracts may be due to their low content of fat and high levels of amino acids such as arginine, leucine, and isoleucine; the extracts also contain high contents of protein and minerals such as Ca and Fe [3].

Studies on black goat meat have evaluated the quality of loin [1], physicochemical properties [4,5], and methods to extract black goat meat for medicinal use [6,7]. However, there have been limited studies evaluating the nutritional values of different cuts of black goat meat and their antioxidant properties. Therefore, in this study, we evaluated the nutritional value and antioxidant activity of black goat loin (BGL) and black goat rump (BGR) meat cuts.

## MATERIALS AND METHODS

### Preparation of black goat meat

Twenty-four hours post-mortem, BGL and BGR cuts each from three different black goats (14-month-old, male) were obtained from a local meat market. Surface fat and connective tissue were dissected away from each meat sample, and the remaining meat samples were minced finely using a food processor (HMF-3800SS; Hanil, Seoul, Korea). The minced meat samples were stored at −20°C until analysis.

### Nutritional values

#### Evaluation of proximate composition

Proximate compositions of BGL and BGR were evaluated using the Official Methods of Analysis stipulated by the Association of Official Agricultural Chemists (AOAC) [8]. The moisture content was assessed using oven drying of samples at 105°C for 16 h, and the crude protein content was analyzed by the Kjeldahl method using a conversion factor of 6.25. Crude fat was assayed by solvent extraction, and crude ash was analyzed using a furnace at 550°C for 12 h.

#### Evaluation of collagen content

The collagen content was determined by measuring hydroxyproline according to the method of Kim et al [9]. Briefly, each meat sample (5 g) was hydrolyzed with 30 mL of 7 N sulfuric acid for 16 h at 105°C. One milliliter of an acid-hydrolyzed diluted sample was mixed with 0.5 mL of 1.41% chloramine T in a collagen buffer solution (pH 6.0), containing sodium hydroxide (15 g), sodium acetate trihydrate (90 g), citric acid monohydrate (30 g), and 1-propanol (290 mL) per 1 L of water. The mixture was shaken for 20 min at 23°C±1°C, then mixed with 0.5 mL of the reactive color reagent (5 g of 4-dimethylaminobenzaldehyde, 17.5 mL of 60% sulfuric acid, and 32.5 mL of 2-propanol) and incubated in a water bath at 60°C for 15 min. After the reaction was completed, absorbance was measured at 558 nm using a spectrophotometer (SpectraMax M2e; Molecular Devices, Sunnyvale, CA, USA). The hydroxyproline content was calculated using a standard curve, and the collagen content in the samples was calculated using a correction factor of 8.0.

#### Mineral content

To analyze the contents of Fe, Ca, P, K, and Na in BGL and BGR, 2 g of each meat sample was ashed at 550°C. The ashed meat samples were dissolved in 65% nitric acid, and the solutions were transferred into 100-mL volumetric flasks, making up to volume with distilled water. The mineral contents in the diluted solutions were analyzed using an inductively coupled plasma optical emission spectrometer (Optima 7300 DV; PerkinElmer, Schwerzenbach, Switzerland).

#### Fatty acid composition

To determine the fatty acid composition, 2 g of each meat sample was homogenized in 15 mL of Folch solution (chloroform/methyl alcohol, 2:1, v/v) containing 40 μL of *tert*-butyl-4-hydroxyanisole and then filtered through Whatman No. 1 filter paper (Clifton, NJ, USA). Each filtered sample was mixed with 2 mL of 0.88% KCl and shaken vigorously. The separated bottom layer was dried under nitrogen gas, and fatty acid methyl ester (FAME) derivatives were generated via methylation of fatty acids with boron trifluoride in methanol for 1 h at 90°C. FAMEs were analyzed using a gas chromatograph (6890N; Agilent Technologies, Santa Clara, CA, USA) equipped with a CP-Sil 88 capillary column (100 m×0.25 mm id×0.20 μm film thickness; Agilent CP7489, Agilent Technologies, USA) and a flame ionization detector. The initial oven temperature was 150°C, which was then increased to 200°C at a rate of 7°C/min, held at 200°C for 20 min, increased to 250°C at a rate of 3°C/min, and held at 250°C for 5 min. The injector and detector temperatures were 260°C and 280°C, respectively. Helium was used as a carrier gas at a flow rate of 1 mL/min, and 1 μL of each sample was injected at a splitting ratio of 1:100. Each fatty acid was identified via matching its retention time with that of a respective standard using a commercially available mixture of fatty acids (PUFA No. 2-Animal Source; Supelco, Bellefonte, PA, USA).

#### L-carnitine content

The L-carnitine content was analyzed in the meat samples according to the method of Shimada et al [10], with slight modifications. Briefly, 5 g of each sample was homogenized in 25 mL of 0.3 M perchloric acid (PCA) on ice. After centrifugation at 1,140×*g* for 10 min, the supernatant was filtered through a glass microfiber filter (GF/C; Whatman, USA), and then the pellet was re-extracted with 20 mL of 0.3 M PCA. The volume of the pooled supernatants was adjusted to 50 mL with 0.3 M PCA. One milliliter of the extract was neutralized with 1.2 M K_2_CO_3_. After centrifugation at 8,385×*g* for 10 min, the supernatant was filtered through a 0.45-μm membrane filter. The standard or sample (50 μL) was loaded with 50 μL of a reaction buffer, containing 0.93 mM 5,5′-dithiobis-(2-nitrobenzoic acid), 0.55 mM acetyl-CoA, 3.05 mM ethylenediaminetetraacetic acid, and 610 mM Tris–HCl, pH 7.5, in a 96-well microplate. The blank absorbance of the samples was measured at 415 nm using a SpectraMax M2e spectrophotometer (Molecular Devices, USA) after incubation at 37°C for 10 min. For the enzymatic reaction, 25 μL (0.5 U) of carnitine acetyltransferase (EC 2.3.1.7; Sigma, St. Louis, MO, USA) was added, and the mixture was incubated at 37°C for 30 min. The final absorbance was measured at 415 nm, and the difference between the blank and final absorbance was used to calculate the content of L-carnitine. L-carnitine was purchased from Sigma Co. (USA) to generate a standard curve.

#### Creatine, creatinine, and dipeptide contents

Creatine, creatinine, and dipeptide (carnosine and anserine) contents were measured using the method of Mora et al [11] with slight modifications. Briefly, 2.5 g of a meat sample was homogenized with 7.5 mL of 0.01 N HCl, and the homogenate was centrifuged at 3,000×*g* for 30 min. The supernatant was filtered through a glass microfiber filter (GF/C; Whatman, USA), and then 250 μL of the filtrate was deproteinized by incubation with 750 μL of acetonitrile for 20 min at 4°C. The sample was centrifuged at 10,000×*g* for 10 min, and the supernatant was filtered through a 0.22-μm membrane filter before analysis. The contents of creatine, creatinine, and dipeptides (carnosine and anserine) were analyzed using a liquid chromatograph (1260 Infinity; Agilent Technologies, USA), equipped with an Atlantis HILIC silica column (4.6×150 mm×3 μm; Waters, Milford, MA, USA), at the column temperature of 35°C. Mobile phase A was 0.65 mM ammonium acetate (pH 5.5) in water/acetonitrile (25:75, v/v), and mobile phase B was 4.55 mM ammonium acetate (pH 5.5) in water/acetonitrile (70:30, v/v). We used a linear gradient of phase B from 0% to 100% over 13 min at a flow rate of 1.4 mL/min. Creatine, carnosine, and anserine were detected at 214 nm, and creatinine was detected at 236 nm using a diode array detector (Agilent Technologies, USA). The contents were calculated using a standard curve generated for each respective standard (Sigma, USA).

### Antioxidant activities

#### Preparation of meat samples for determining antioxidant activities

To determine the antioxidant activities, 4 g of each meat sample was homogenized in 20 mL of distilled water. The homogenates were filtered through Whatman No. 4 filter paper (USA), followed by the addition of 4 mL of chloroform and vortexing. The bottom layer was lyophilized and stored at −20°C until use.

#### Ferric reducing antioxidant power activity

The ferric reducing antioxidant power (FRAP) assay was carried out according to the method of Benzie and Strain [12], with slight modifications. The FRAP working solution contained 300 mM acetate buffer (pH 3.6), 10 mM 2,4,6-tripyridyl-S-triazine in 40 mM HCl, and a 20 mM FeCl_3_·6H_2_O solution at a ratio of 10:1:1 (v/v/v), respectively. Each meat extract (25 μL) was reacted with 175 μL of the FRAP working solution for 30 min at 37°C in the dark. The absorbance of the reaction solution was determined at 590 nm using a spectrophotometer (Molecular Devices, USA). The standard curve was obtained using Trolox (Sigma, USA). The results were expressed as micromoles of Trolox equivalent (TE) per gram of dry matter (DM).

#### 2,2-Azinobis (3-ethyl-benzothiazoline-6-sulfonic acid) radical-scavenging activity

To analyze the 2,2-azinobis (3-ethyl-benzothiazoline-6-sulfonic acid) (ABTS) radical-scavenging activity, we used the method of Re et al [13] with some modifications. To generate the ABTS^+^ radical, a stock solution was made by mixing equal volumes of a 14 mM ABTS^+^ solution and a 5.9 mM potassium persulfate solution; the resulting solution was then reacted at 23°C±1°C for 12 h in the dark. The stock solution was diluted with distilled water to an absorbance of 0.700±0.02 at 735 nm, as assessed using a spectrophotometer (Molecular Devices, USA) at 30°C. Meat extracts (50 μL) were reacted with 950 μL of the ABTS^+^ radical solution for 30 min at 30°C in the dark. The absorbance of the reaction solution was determined at 735 nm, and the results were expressed as micromoles of TE per gram of DM.

#### Oxygen radical absorption capacity

The oxygen radical absorption capacity (ORAC) assay was performed according to the method of Gillespie et al [14], with some modifications, using 75 mM potassium phosphate buffer (pH 7.4) at 37°C. First, 25 μL of a meat extract was mixed with 150 μL of 80 nM fluorescein. After incubation at 37°C for 15 min, 25 μL of 150 mM 2,2′-azobis (2-amidinopropane) hydrochloride (AAPH) was added to generate peroxyl radicals. The change in the absorbance of the reaction was recorded every minute at an excitation wavelength of 480 nm and emission wavelength of 520 nm at 37°C using a SpectraMax M2e spectrophotometer (Molecular Devices, USA). Trolox was used as a standard, and the results were expressed as micromoles of TE per gram of DM.

### Statistical analysis

Statistical analysis was performed using the SAS program version 9.4 (SAS Institute, Inc., Cary, NC, USA). Data comparison was performed using a Student’s *t*-test. Differences among means were considered significant at p<0.05. All data are expressed as the mean±standard error of the mean.

## RESULTS AND DISCUSSION

### Proximate compositions and collagen and mineral contents of black goat loin and black goat rump

The proximate compositions and collagen and mineral contents of BGL and BGR are shown in Table 1. BGL and BGR contained 75.00% and 75.49% moisture, 21.60% and 21.30% crude protein, 1.48% and 1.40% crude fat, and 1.41% and 1.25% ash, respectively, with no significant differences between the cuts. Similarly, Choi et al [15] have reported that the proximate composition of black goat meat included 74.40% to 76.04% moisture, 19.83% to 20.47% crude protein, 1.64% to 3.56% crude fat, and 1.04% to 1.11% crude ash. Hwang et al [16] have reported that the crude fat content of black goat meat was between 1.12% and 1.59%, which agrees with our results. On the other hand, Kim et al [5] have reported that the crude fat content of the BGL meat was 7.81% to 9.43% at the different age of castration, which was higher than our values. Another study has reported that the proximate composition of goat meat included 67.0% to 75.2% moisture, 18.9% to 24.8% crude protein, 3.25% to 12.6% crude fat, and 0.95% to 1.19% crude ash, depending on the breed [17].

The collagen content of BGR was higher than that of BGL (p<0.05), which may be due to higher exercise status of the rump muscle compared with that of the loin muscle in black goats. Our data are similar to the results of Cho et al [18] showing that the beef rump showed a higher collagen content than the loin did because of the difference in exercise strength between them. Generally, the goat meat has a higher content and lower solubility of collagen compared with those in the sheep meat, which is linked to lower tenderness of goat meat compared with that of sheep meat and may explain the lower consumer preference for goat meat [1].

Minerals are essential for maintaining the normal growth and a good health. Lean meat is an excellent source of minerals. The feed type and breed of the animal can affect the mineral content of meat. The Fe, Ca, P, K, and Na contents in BGL and BGR are shown in Table 1. Red meat can contain a high level of bioavailable Fe, which is abundant in red meats such as lean beef (2.72 mg/100 g), lamb (1.74 mg/100 g), veal (1.11 mg/100 g), and goat (4.37 mg/100 g) [19]. In this study, the Fe content in BGR (1.48 mg/100 g) was significantly (p<0.05) higher than that in BGL (1.35 mg/100 g). Jeong et al [3] have reported that the Fe content in black goat meat was 0.6 mg/100 g, which is lower than that observed in our study. Ca and P are major minerals required for bone development, and the ideal Ca/P ratio, based on the recommended daily allowance of nutrients, is between 1 and 2 [19]. In this study, the Ca/P ratio in both BGL and BGR (1.54 and 1.82, respectively) satisfied the recommended criteria, while being higher in BGR than in BGL (p<0.05). Fresh meat is also a rich source of K, and its contents in beefsteak, mutton, and goat meat were shown to be 334, 240, and 350 mg/100 g, respectively [19]. In this study, the K content in BGL (325.22 mg/100 g) was higher than that in BGR (281.40 mg/100 g; p<0.05). BGR showed a higher content of Na than BGL did (p<0.05).

### Fatty acid compositions of black goat loin and black goat rump

The fatty acid composition of meat can influence its organoleptic properties and quality. The fatty acid compositions of BGL and BGR are shown in Table 2. The predominant fatty acids in BGL and BGR were palmitic acid, stearic acid, oleic acid, linoleic acid, and arachidonic acid. The contents of oleic acid and monounsaturated fatty acids (MUFAs) in the meat are positively associated with its organoleptic properties [4]. In this study, the oleic acid contents in BGL and BGR were 30.00% and 28.76%, respectively. These levels were slightly lower than the range of 37.23% to 39.80% previously reported for black goat meat [16], regular goat meat [1], and beef [20]. BGR showed higher contents of myristic acid, palmitic acid, palmitoleic acid, γ-linolenic acid, and docosahexaenoic acid than did BGL (p<0.05), while BGL showed a higher stearic acid content than did BGR (p<0.05). However, there were no significant differences in the levels of total saturated fatty acids (SFA) and unsaturated fatty acids (UFAs) between BGR and BGL. The polyunsaturated fatty acid (PUFA)/SFA ratio is used to evaluate the nutritional quality of fat in meat, and the recommended value is more than 0.4 or 0.5 [21]. In addition, the American Heart Association recommends a fatty acid balance of SFAs, MUFAs, and PUFAs of approximately 1:1.5:1. In this study, BGL and BGR contained 39.07% and 40.13% SFAs and 22.37% and 22.89% PUFAs, respectively, showing the same PUFA/SFA ratio of 0.57, as compared with the previously reported ranges of 0.31 to 0.37 for goat loin meat and 0.31 to 0.49 for goat rump meat [22]. BGL and BGR also showed a higher PUFA/SFA ratio than beef did at 0.13 [20]. High dietary intake of long-chain SFAs, except stearic acid, has been associated with an increase in plasma cholesterol levels in humans [23]. Oleic acid is known to reduce cholesterol by suppressing low-density lipoprotein cholesterol and triglycerides, and stearic acid is partially converted to oleic acid *in vivo* [23]. Therefore, desirable fatty acids (DFAs), which represent a total of stearic acid and all UFAs, are considered another factor of nutritional quality of fat for humans because of their ability to reduce plasma cholesterol levels [22]. Generally, the DFA content ranges in goat meat from 61% to 80% [22]. In this study, BGL showed a higher DFA content than did BGR (77.27% and 74.81%, respectively; p<0.05), and both values were higher than those reported for beef and lamb (63.80% and 61.58%, respectively) [20]. Therefore, black goat meat can provide a balanced content of fatty acids for human consumption.

### Bioactive compounds in black goat loin and black goat rump

The bioactive compounds (L-carnitine, creatine, creatinine, carnosine, and anserine) found in BGL and BGR are shown in Table 3. As a ubiquitous constituent of mammalian plasma, supplied by dietary meat and dairy products [10], L-carnitine supports the transport of long-chain fatty acids from the outer to the inner mitochondrial membrane, allowing β-oxidation of long-chain fatty acids [24]. The level of L-carnitine can be considered a major factor in cellular energy production using long-chain fatty acids. Generally, red meat is known as an abundant source of L-carnitine. In this study, the BGL and BGR meats contained L-carnitine at 1.37 and 1.25 μmol/g wet tissue, respectively. According to Shimada et al [10], the content of L-carnitine in fresh semitendinosus muscles of cattle varies from 1.67 to 3.57 μmol/g wet tissue.

Creatine and its phosphorylated form creatinine are impor tant for energy production in muscles, and supplementation with creatine can help enhance the muscle performance [25]. In our study, BGL contained creatine at 187.87 mg/100 g, which was significantly higher than its content in BGR (178.26 mg/100 g). Meanwhile, the creatinine content was 3.13 and 2.97 mg/100 g in BGL and BGR, respectively, showing no significant difference. The creatine and creatinine contents have been reported to vary in animals, as follows: pork loin (427.32 and 5.71 mg/100 g, respectively) [11], chicken breast (336.24 and 1.78 mg/100 g, respectively) [11], and beef (526 and 21 mg/100 g, respectively) [25].

Carnosine and anserine are dipeptides, which provide a buffering capacity in skeletal muscles. Carnosine shows antiglycation activity and antioxidant properties, such as scavenging of reactive oxygen species and chelating metal ions [25]. Anserine functions as an antioxidant in humans by chelating Cu [25]. In this study, the carnosine and anserine contents of BGL (62.25 and 81.93 mg/100 g, respectively) were higher than those of BGR (49.54 and 66.32 mg/100 g, respectively; p<0.05). Mora et al [11] have reported that the contents of carnosine, creatine, and creatinine in different muscles of swine were primarily affected by the muscle type. Glycolytic muscles such as type 2A and type 2B show high contents of carnosine, creatine, and creatinine. Similarly, in this study, BGL (glycolytic muscle) showed a higher content of carnosine, creatine, and creatinine than did BGR (p<0.05). The carnosine and anserine contents were 462 and 10.76 mg/100 g [11] and 372 and 67 mg/100 g [25] in pork loin and beef, respectively. Thus, the carnosine and anserine contents vary in different species. Generally, red meats such as pork and beef have a high carnosine-to-anserine ratio, 42.9 [11] and 5.5 [25], respectively. Interestingly, in this study, the carnosine-to-anserine ratios in BGL and BGR, another type of red meat, were 0.80 and 0.75, respectively, i.e., BGL and BGR contained more anserine than carnosine. This pattern is similar to that found in rabbit and red deer meat (ratios of 0.44 and 0.73, respectively) [26]. These results suggested that BGL could provide more bioactive compounds than BGR could, and both BGL and BGR showed similar carnosine/anserine ratios to those found in rabbits and red deer.

### Antioxidant activities of black goat loin and black goat rump

The antioxidant activities of BGL and BGR are shown in Table 4. The FRAP, ABTS, and ORAC methods are often used to evaluate the antioxidant activity of foods. The FRAP method is based on the reduction of a ferroin analog and can measure the total reducing capacity [27]. The ABTS method is used to measure the ability of hydrogen-donating antioxidants to scavenge the ABTS^+^ radical cation generated in the aqueous phase [27]. The ORAC method is used to measure the antioxidant activity against peroxyl radicals generated by AAPH and is the most biologically relevant method for analyzing antioxidant activity [28]. Intake of antioxidants helps maintain an adequate antioxidant status and prevent cellular damage from oxidative stress generated in the body [28]. In our study, the FRAP, ABTS, and ORAC values for BGL and BGR were 15.92 and 15.92, 12.51 and 12.90, and 101.25 and 99.06 μmol TE/g DM, respectively, showing that there was no significant difference in the antioxidant activity between BGL and BGR. Mirzaei et al [29] have reported that goat meat had 42% diphenyl-picrylhydrazyl radical-scavenging activity. In our previous study, boiled pork meat showed a FRAP activity of 3.66 to 5.31 μmol TE/g DM, ABTS activity of 26.60 to 39.43 μmol TE/g DM, and an ORAC activity of 143.74 to 198.35 μmol TE/g DM [30]. Hanwoo beef showed an ORAC activity of 145.54 μmol TE/g DM [31]. These results indicate that black goat meat possesses a high reducing power and can help maintain an adequate antioxidant status in humans.

In this study, we evaluated nutritional values and antioxi dant activities of BGL and BGR. In particular, BGL showed higher contents of DFAs, creatine, creatinine, anserine, and carnosine than did BGR. However, BGR had higher collagen and mineral contents, especially with respect to the Ca/P ratio, than did BGL. Although there was no significant difference in the antioxidant activity between BGL and BGR, both goat meat cuts showed a higher reducing power activity than did boiled pork meat [30] assessed in previous studies. This work was a novel study comparing nutritional values and antioxidant activities of black goat meat with respect to the cut. Therefore, our results help elucidate the antioxidant properties of fresh black goat meat, which is used as a nutritional food source in human consumption.

## Figures and Tables

**Table 1:** Proximate compositions and collagen and mineral contents of different meat cuts from black goats

Traits	Cuts		SEM

	BGL	BGR	
Proximate composition (%)			
Moisture	75.00^a^	75.49^a^	0.281
Crude protein	21.60^a^	21.30^a^	0.743
Crude fat	1.48^a^	1.40^a^	0.071
Crude ash	1.41^a^	1.25^a^	0.076
Collagen (g/100 g)	0.59^b^	1.12^a^	0.010
Minerals (mg/100 g)			
Fe	1.35^b^	1.48^a^	0.003
Ca	5.22^b^	6.09^a^	0.029
P	3.39^a^	3.34^a^	0.015
K	325.22^a^	281.40^b^	0.700
Na	76.03^b^	94.97^a^	0.215
Ca:P	1.54^b^	1.82^a^	0.008

BGL, black goat loin; BGR, black goat rump; SEM, standard error of the mean.

a,b Means with different superscript letters within a row are significantly different at p<0.05.

**Table 2:** Fatty acid compositions of different meat cuts from black goats

Fatty acid (%)	Cuts		SEM

	BGL	BGR	
C14:0 (myristic acid)	1.37^b^	2.14^a^	0.079
C16:0 (palmitic acid)	21.36^b^	23.05^a^	0.302
C16:1n7 (palmitoleic acid)	0.88^b^	1.22^a^	0.031
C18:0 (stearic acid)	16.35^a^	14.94^b^	0.222
C18:1n9 (oleic acid)	30.00^a^	28.76^a^	0.328
C18:1n7 (vaccenic acid)	5.84^a^	5.18^a^	0.312
C18:2n6 (linoleic acid)	10.48^a^	11.64^a^	0.359
C18:3n6 (γ-linolenic acid)	0.07^b^	0.08^a^	0.003
C18:3n3 (α-linolenic acid)	0.15^a^	0.18^a^	0.008
C20:1n9 (eicosenoic acid)	1.84^a^	1.83^a^	0.206
C20:4n6 (arachidonic acid)	9.88^a^	9.04^a^	0.431
C20:5n3 (eicosapentaenoic acid)	0.35^a^	0.30^a^	0.019
C22:4n6 (adrenic acid)	1.43^a^	1.59^a^	0.055
C22:6n3 (docosahexaenoic acid)	0.00^b^	0.05^a^	0.000
SFA	39.07^a^	40.13^a^	0.350
UFA	60.93^a^	59.87^a^	0.350
MUFA	38.56^a^	36.99^a^	0.541
PUFA	22.37^a^	22.89^a^	0.860
MUFA/SFA	0.99^a^	0.92^b^	0.008
PUFA/SFA	0.57^a^	0.57^a^	0.026
DFA	77.27^a^	74.81^b^	0.358

Data are the means (n = 3).

BGL, black goat loin; BGR, black goat rump; SEM, standard error of the mean; SFA, saturated fatty acid; UFA, unsaturated fatty acid; PUFA, polyunsaturated fatty acid; MUFA, monounsaturated fatty acid; DFAs, desirable fatty acids (stearic acid+UFAs).

a,b Means with different superscript letters within a row are significantly different at p<0.05.

**Table 3:** Bioactive compounds in different meat cuts from black goats

Traits	Cuts		SEM

	BGL	BGR	
L-carnitine1)	1.37^a^	1.25^a^	0.041
Creatine2)	187.87^a^	178.26^b^	1.342
Creatinine	3.13^a^	2.97^b^	0.023
Carnosine	65.25^a^	49.54^b^	1.481
Anserine	81.93^a^	66.32^b^	1.825
Carnosine:anserine	0.80^a^	0.75^b^	0.002

BGL, black goat loin; BGR, black goat rump; SEM, standard error of the mean.

1) The L-carnitine content is expressed in μmol/g wet tissue.

2) Creatine, creatinine, carnosine, and anserine contents are expressed in mg/100 g wet tissue.

a,b Means with different superscript letters within a row are significantly different at p<0.05.

**Table 4:** Antioxidant activities of different meat cuts from black goats

Antioxidant activities (μmol TE/g DM)	Cuts		SEM

	BGL	BGR	
FRAP	15.92	15.92	0.210
ABTS	12.51	12.90	0.216
ORAC	101.25	99.06	1.231

TE, Trolox equivalent; DM, dry matter; BGL, black goat loin; BGR, black goat rump; SEM, standard error of the mean; FRAP, ferric reducing antioxidant power; ABTS, 2,2-azinobis (3-ethyl-benzothiazoline-6-sulfonic acid); ORAC, oxygen radical absorption capacity.
